# Analysis of the microbiome in maternal, intrauterine and fetal environments based on 16S rRNA genes following different durations of membrane rupture

**DOI:** 10.1038/s41598-023-41777-z

**Published:** 2023-09-11

**Authors:** Huifen Yin, Jiao Yu, Wei Wu, Xiaotian Li, Rong Hu

**Affiliations:** The Obstetrics and Gynecology Hospital of Fudan University, 419 Fangxie Road, Shanghai, 200011 China

**Keywords:** Microbiology, Diseases, Risk factors

## Abstract

The incidence of chorioamnionitis and neonatal sepsis increases with the increasing time of rupture of membranes. Changes in the amount and categories of microbiomes in maternal and fetal environments after membrane rupture have yet to be discussed. In order to determine the microbiome diversity and signature in the maternal, intrauterine, and fetal environments of different durations following membrane rupture, we collected samples of fetal membrane, amniotic fluid, cord blood and maternal peripheral blood from singleton pregnant women and divided them into five groups according to the duration of membrane rupture. DNA was isolated from the samples, and the V3V4 region of bacterial 16S rRNA genes was sequenced. We found that the alpha diversity of the fetal membrane microbiome increased significantly 12 h after membrane rupture, while the beta diversity of the amniotic fluid microbiome increased 24 h after membrane rupture. In cord blood, the mean proportion of *Methylobacterium* and *Halomonadaceae* reached the highest 12 h after membrane rupture, and the mean proportion of *Prevotella* reached the highest 24 h after membrane rupture. The LEfSe algorithm showed that *Ruminococcus*, *Paludibaculum*, *Lachnospiraceae*, and *Prevotella* were detected earlier in cord blood or maternal blood and then detected in fetal membranes or amniotic fluid, which may suggest a reverse infection model. In conclusion, the microbes may invade the placenta 12 h after membrane rupture and invaded the amniotic cavity 24 h after membrane rupture. In addition to the common ascending pattern of infection, the hematogenous pathway of intrauterine infection should also be considered among people with rupture of membranes.

## Introduction

Intrauterine infection is associated with 40–70% of spontaneous preterm births and 1–13% of term births, which can manifest as both clinical and recessive infections^1^. It may cause neonatal periventricular leukomalacia, cerebral palsy, respiratory distress, premature neonatal sepsis, bronchopulmonary dysplasia, and necrotizing enterocolitis^1,2^. The association between prolonged rupture of membranes and intrauterine infection has long been recognized. The incidence of premature rupture of membranes (PROM) at term and before term is approximately 8% and 3%, respectively. Approximately 1/3 of pregnant women with preterm premature rupture of membranes (pPROM) may have severe infections, such as chorioamnionitis and umbilical cord inflammation, endometritis or sepsis^3^. The incidence of chorioamnionitis and neonatal sepsis at term delivery also increases with the increasing duration of ruptured membranes. The incidence of chorioamnionitis increased from 2.7 to 11.8% when the length of rupture of membranes exceeded 12 h^4^; the rate of neonatal sepsis was 0.3% at a membrane rupture to delivery interval below 6 h, 0.5% at 6–18 h, 0.8% at 18–24 h, and 1.1% after 24 h^5^.

Recent improvements in 16S rRNA gene amplicon high-throughput sequencing have enabled researchers to further identify intrauterine-related bacteria and their possible origins^6–9^. Some studies have suggested that instead of being sterile, uterine tissue may harbor its own microbiome^7–10^. In addition, the composition of intrauterine microbial communities is not always static. It was reported that the average concentration of prokaryotic DNA after membrane rupture was more than 10 times higher than that before membrane rupture, indicating that most microbial invasion started after uterine contraction and membrane rupture^6^. Although it has been suggested that the incidence of chorioamnionitis and neonatal sepsis increases with the increasing time of rupture of membranes, studies have revealed microbial signatures among different durations following rupture of the fetal membrane in maternal, intrauterine, and fetal environments, and their relationship is lacking. Changes in the amount and categories of microbiomes in maternal and fetal environments after membrane rupture have yet to be discussed. In addition to the common ascending pattern of infection, other sources of infection may also exist. Therefore, to provide evidence for clinicians to make timely interventions after membrane rupture, there were two objectives of this study: first, to determine the microbiome diversity and signature in the maternal, intrauterine, and fetal environments with different durations following membrane rupture to delivery; second, to analyze their probable transmission pathways using a metagenomic approach based on 16S rRNA genes.

## Results

### Cohort demographics

A total of 102 singleton gravida were involved, and 264 samples were collected in this study. The demographic characteristics of the five groups are illustrated in Table 1. The average gestational weeks at delivery and fetal weight of women with a duration of membrane rupture of more than 48 h were significantly different from those of women with a duration of less than 6 h. There were no differences between the groups in terms of other maternal and neonatal characteristics. Specimen types included cord blood (102 samples), fetal membranes (92 samples), amniotic fluid (28 samples), and maternal peripheral blood (42 samples). The exact number of samples in each group is shown in Table 2.

**Table 1:** Demographic characteristics of women in different groups.

Duration of membrane rupture	< 6 h (n = 23)	6 to < 12 h (n = 24)		12 to < 24 h (n = 26)		24 to < 48 h (n = 18)		≥ 48 h (n = 11)	
	Mean ± sd/n (%)	Mean ± sd/n (%)	p	Mean ± sd/n (%)	p	Mean ± sd/n (%)	p	Mean ± sd/n (%)	p
Maternal condition									
Maternal age (years)	29.8 ± 2.4	29.0 ± 3.7	0.37	31.0 ± 3.4	0.19	29.9 ± 3.7	0.95	29.2 ± 4.3	0.58
Times of gestation	1.4 ± 0.7	1.8 ± 1.2	0.22	1.6 ± 0.8	0.39	1.9 ± 1.3	0.10	2.0 ± 1.3	0.16
Times of parity	0.1 ± 0.3	0.0 ± 0.2	0.29	0.0 ± 0.0	0.08	0.2 ± 0.4	0.75	0.1 ± 0.3	0.74
Gestational age at delivery (weeks)	39.0 ± 1.7	38.5 ± 2.0	0.39	38.7 ± 1.5	0.51	37.9 ± 4.5	0.31	34.7 ± 6.0*	0.04*
Count of WBC (10^9^/L)	8.8 ± 2.3	10.0 ± 2.5	0.08	10.1 ± 3.7	0.15	9.9 ± 3.5	0.23	10.0 ± 2.9	0.19
Percentage of N (%)	74.1 ± 5.9	73.9 ± 7.5	0.90	76.9 ± 6.2	0.11	72.4 ± 10.4	0.52	71.9 ± 10.9	0.44
Preterm birth, n (%)	3 (13.0)	4 (16.7)	0.73	3 (11.5)	0.87	2 (11.1)	0.85	4 (36.4)	0.12
Maternal infection, n (%)	0 (0)	1 (4.2)	0.32	3 (11.5)	0.09	4 (22.2)	0.02*	1 (9.1)	0.14
Antibiotic administration, n (%)	4 (17.4)	5 (20.8)	0.76	8 (30.8)	0.28	5 (27.8)	0.43	4 (36.4)	0.22
Meconium stained amniotic, n (%)	3 (13.0)	1 (4.2)	0.28	2 (7.7)	0.54	1 (5.6)	0.42	1 (9.1)	0.74
Cesarean section, n (%)	6 (26.1)	7 (29.2)	0.81	10 (38.5)	0.36	8 (44.4)	0.22	7 (63.6)	0.04*
GDM, n (%)	1 (4.3)	3 (12.5)	0.32	2 (7.7)	0.63	2 (11.1)	0.41	0 (0.0)	0.48
HDP, n (%)	0 (0)	2 (8.3)	0.16	0 (0)	–	0 (0)	–	1 (9.1)	0.14
FGR, n (%)	0 (0)	0 (0)	–	0 (0)	–	0 (0)	–	0 (0)	–
Neonatal condition									
Birth weight (g)	3287 ± 467	3176 ± 453	0.41	3357 ± 504	0.62	3176 ± 794	0.58	2576 ± 1091	0.07
Apgar score at 1 min < 7	0 (0)	0 (0)	–	1 (3.8)	0.34	1 (5.6)	0.25	1 (9.1)	0.14
Apgar score at 5 min < 7	0 (0)	0 (0)	–	0 (0)	–	1 (5.6)	0.25	1 (9.1)	0.14
Admission to neonatal ward, n (%)	3 (13.0)	4 (16.7)	0.73	5 (19.2)	0.56	4 (22.2)	0.44	3 (27.3)	0.31
Neonatal infection, n (%)	1 (4.3)	1 (4.2)	0.96	1 (3.8)	0.93	2 (11.1)	0.41	3 (27.3)	0.05
Perinatal mortality, n (%)	0 (0)	0 (0)	–	0 (0)	–	1 (5.6)	0.25	1 (9.1)	0.14

*Compared with < 6 h group, p ≤ 0.05.

*WBC* white blood cells, *N* neutrophilic granulocyte, *GDM* gestational diabetes mellitus, *HDP* hypertensive disorders of pregnancy, *FGR* fetal growth restriction.

**Table 2:** Numbers of samples in each specimen of different durations of membrane rupture.

Duration of membrane rupture (h)	Fetal membrane n = 92	Amniotic fluid n = 28	Cord blood n = 102	Maternal peripheral blood n = 42
< 6	23	3	23	11
6 to < 12	22	6	24	6
12 to < 24	22	6	26	11
24 to < 48	16	7	18	9
≥ 48	9	6	11	5

### Survey of reproductive microbiome content

**Figure 1:**
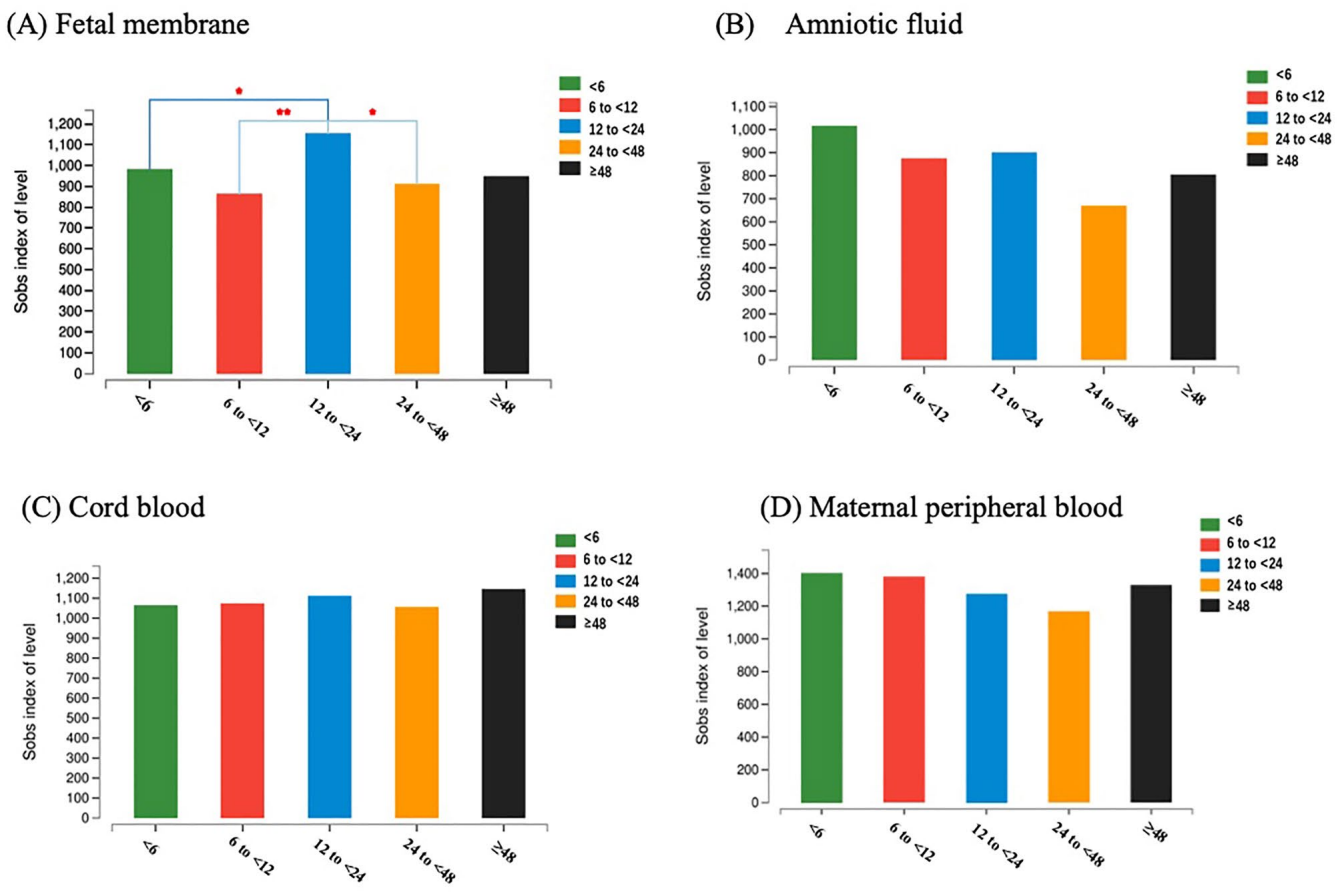
Alpha diversity analysis of bacterial 16S rRNA genes was calculated at the OTU level to reflect the differentiation among the duration following rupture of membranes in the fetal membranes, amniotic fluid, cord blood and maternal peripheral blood. The Sobs index of OTU-level abundance (y-axis) is represented by a vertical bar, where each bar indicates a group by the duration following rupture of membranes. In the fetal membrane, the 12 to < 24 h group was more diverse than the < 6, 6 to < 12 and 24 to < 48 h groups according to Wilcoxon’s test.

Alpha diversity was calculated to reflect the “within” community richness of the microbial community for each sample. In the fetal membrane, the microbiotas of the 12 to < 24 h group were found to be more diverse than the bacterial communities found in the < 6, 6 to < 12 and 24 to < 48 h groups when compared in Wilcoxon’s test (Fig. 1A). There was no significant difference in the microbiome of amniotic fluid, cord blood or maternal peripheral blood (Fig. 1B–D).

**Figure 2:**
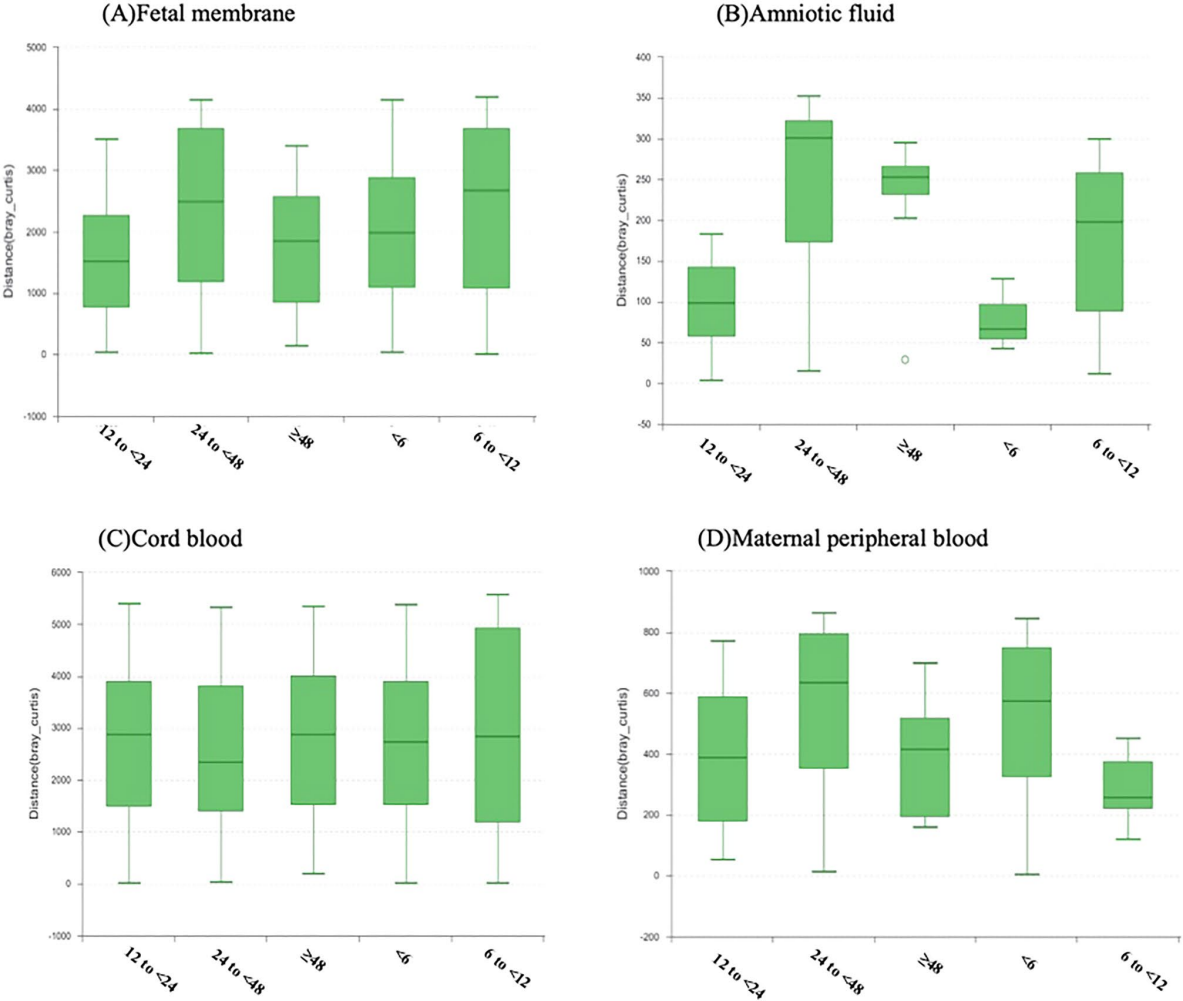
Beta diversity analysis of bacterial 16S rRNA genes was calculated at the OTU level to reflect the differentiation among the duration following rupture of membranes in the fetal membranes, amniotic fluid, cord blood and maternal peripheral blood. The distance calculated on the OTU level in Bray‒Curtis (y-axis) is represented by a vertical bar, where each bar indicates a group by the duration following rupture of membranes. In amniotic fluid, the microbiome of the 24 to < 48 h and ≥ 48 h groups were more diverse than that of the < 6 and 12 to < 24 h groups.

Beta diversity was calculated to reflect the overall microbiota dissimilarities in samples of different groups. In amniotic fluid, the microbiome of the 24 to < 48 and ≥ 48 h groups were more diverse than that of the < 6 and 12 to < 24 h groups (Fig. 2B). There was no statistically significant difference in the microbiome of the fetal membrane, cord blood or maternal peripheral blood between the different groups (Fig. 2A,C,D).

### Microbial features associated with the duration of membrane rupture

**Figure 3:**
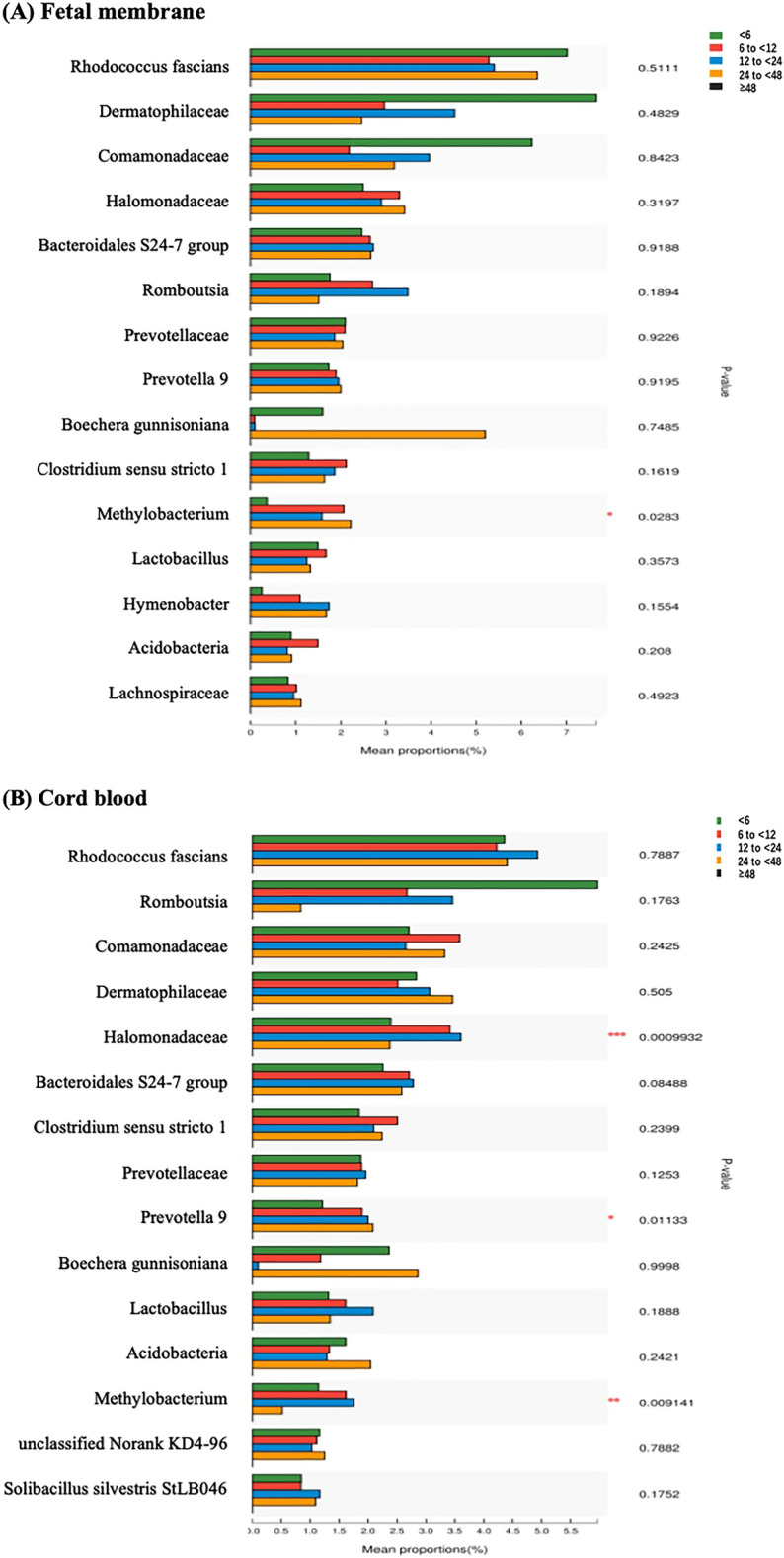
Differentiation of microbe abundance (mean proportion > 1%) at the genus level among the different durations following rupture of the membrane in the Kruskal‒Wallis test. The y-axis represents bacteria at the genus level, and the x-axis represents their mean proportion. (**A**) Comparison in fetal membranes, (**B**) comparison in cord blood.

We compared the bacteria (mean proportion > 1%) at the genus level among different durations of membrane rupture using the Kruskal‒Wallis test and found diversity. The results showed that the mean proportion of *Prevotella* and *Halomonadaceae* in cord blood, *Methylobacterium* in both fetal membranes and cord blood, was different between the five groups (Fig. 3A,B). There was no significant difference between different durations of membrane rupture in amniotic fluid and maternal peripheral blood samples.

To identify the specific taxonomic biomarkers in each tissue of different membrane rupture durations, we additionally applied the linear discriminant analysis effect size (LEfSe) algorithm to identify microbial species that were significantly more prevalent in each period. The specific microbes are shown in Table 3. *Ruminococcus, Paludibaculum, Lachnospiraceae, and Prevotella* were detected earlier in the fetal membrane and amniotic fluid and then detected in cord blood, while *Rhodobium, Christensenellaceae, Nonomuraea, Thauera*, and *Pseudonocardia* were detected earlier in cord blood or maternal blood than in the fetal membrane and amniotic fluid.

**Table 3:** The differential bacteria among the duration following rupture of membranes in the fetal membranes, amniotic fluid, cord blood and maternal peripheral blood.

Duration of membrane rupture(h)	Fetal membrane	Amniotic fluid	Cord blood	Maternal peripheral blood
< 6	Ruminococcus*, Blautia, Patulibacter, Dokdonella, Lachnospira, Geodermatophilus, Eubacterium, Bifidobacterium, Xanthomonadales, Lachnospira, Paludibaculum*, Sporichthyaceae, Rhodospirillales, Sphaerisporangium, Dongia	Blastococcus, Desulfurellaceae, lamia, Gaiella, Myxococcales, Bdellovibrio, Hyphomicrobium, Bryobacter, Phyllobacteriaceae, Bifidobacterium, Pseudocatenulatum, Anoxybacillus flavithermus, Ruminococcus*, Filimonas estrogen degrading bacterium	*Rikenellaceae****,**** Rhodobium*^+^*, bacillales*	*Bacteroides plebeius, Christensenellaceae*^+^*, Legionellales, Coxiellaceae, Nesterenkonia, Nitrolancea, Sphaerobacteraceae, Blautia*
6 to < 12	*Xanthomonadales Incertae Sedis, Mesorhizobium, Niabella, Taibaiella, Labrys, Dechloromonas, Flavitalea, Methylocystaceae, Rudaea, Coriobacteriaceae, Sphingopyxis, Mesorhizobium amorpha, Comamonas testosteroni, Denitratisoma, Legionellaceae*	*Micrococcales, Halomonas, Oscillibacter, Fusobacterium, Ramlibacter*	*Azotobacter beiierinckii, Nonomuraea*^+^*, Cellulomonadaceae*	*Psychrobacter, Xanthobacteraceae, Oligoflexales, Plasticicumulans lactativorans, Tyzzerella, Blastocatellaceae Subgroup4*
12 to < 24	Gemmatimonadaceae, Frankiales, Acidibacter, Planctomycetaceae, Micromonosporaceae, Candidatus Solibacter, Nitrospira, Desulfurellaceae, Haliangium, Hyphomicrobium, Intestinimonas, Arenimonas, Planococcaceae, Rhodobiaceae, Nannocystaceae, Xanthomonadales Incertae Sedis, Chthonomonas, Pseudolabrys, Dorea, Lachnospiraceae*, Roseburia intestinalis, Solirubrobacter	*Bacillus, Streptosporangiaceae, Nonomuraea*^+^*, Roseiflexus, Allobaculum, Gemmatimonadaceae, Desulfurellaceae, Flavobacterium, Acidobacteriaceae, Niabella*	*Thauera*^+^*, Alteromonadales*	*Clostridiaceae, Arthrobacter, Alteromonadales*
24 to < 48	*Phycicoccus aerophilus, Brassica nigra black mustard, Alloprevotella, Arcobacter, Hydrogenophilaceae, Acetoanaerobium*	Brevundimonas nasdae, Prevotella*, Peptococcaceae	*Pseudonocardia*^+^, Lachnospiraceae*	*Desulfovibrio desulfuricans, Limnobacter, Blastocatellaceae Subgroup4*
> or = 48	*Methylobacterium adhaesivum, Micropruina, Thauera*^+^*, Ottowia, Christensenellaceae*^+^*, Paenarthrobacter, Gemmatimonadaceae, Intrasporangiaceae, Ruminococcaceae UCG 014, Syntrophobacter, Acetobacteraceae, Moraxella osloensis, Cytophagaceae bacterium, Enhydrobacter, Solirubrobacterales, Rhodobium*^+^*, Intestinibacter, Pseudonocardiacea*^+^*, Nitrosomonas eutropha, Chthonomonas*	*Bacteroides stercoris3, Aliivibrio finisterrensis, Serinicoccus, Legionella, Pseudolabrys*	Prevotella*, Nitrosomonadaceae, Sulfuritalea, Lactivibrio, Sorangium, Megasphaera, Rickettsiaceae , Parachlamydiaceae, Alkanindiges, Singulisphaera	Rhodobacteraceae, Anaerolinea, Eubacterium xylanophilum, Paludibaculum*, Anaeromyxobacter, Roseomonas lacus, Edaphobacter, Rhizomicrobium, Nocardia, Peptoniphilus, Bacteriovoracaceae, Peredibacter, Ruminococcus*, Nakamurella, Terrabacter, Phyllobacterium, Propionicicella

Bacteria superscripted with * were detected in fetal membrane or amniotic fluid earlier than in cord blood or maternal peripheral blood.

Bacteria superscripted with + were detected in cord blood or maternal peripheral blood earlier than they were detected in fetal membrane or amniotic fluid.

## Discussion

Our 16S-based operational taxonomic unit (OUT) analysis of samples found that the alpha diversity of the fetal membrane microorganisms increased significantly 12 h after membrane rupture, while the beta diversity of the amniotic fluid microbiome significantly increased 24 h after membrane rupture. According to the analysis of the specific bacteria shared among different groups in different tissues, it was found that some bacteria may appear in maternal blood or cord blood earlier, suggesting the possibility of a hematogenous pathway of intrauterine infection. However, further studies should focus on the morbific abundance or duration of these microorganisms to have a better understanding of their association with fetal or neonatal diseases and to minimize the bias of potential contamination.

Our study indicated that the diversity and abundance of microorganisms in amniotic fluid increased 24 h after the rupture of membranes, which was consistent with Seaward's finding that the risk of chorioamnionitis and neonatal infection significantly increased if membrane rupture was over 24 h^11^. Our study also showed that compared with women of membrane rupture time less than 6 h, women of membrane rupture time over 24 h had a higher rate of maternal infection, Although most guidelines recommended expectant management for 24 h after PROM of term births in the absence of signs of infection^12^, a large systemic review found that immediate intervention or intervention within 24 h reduced the risk of maternal infectious morbidity compared with expectant management for PROM at 37 weeks’ gestation or later^13^. Our result may provide evidence for induction of labor earlier after membrane rupture of term or near term births if spontaneous labor does not occur, considering that at least 12–18 h of effective contractions should be allowed for induced labor^14^. Our study also showed that in cord blood, the mean proportion of *Methylobacterium* and *Halomonadaceae* reached the highest 12 h after membrane rupture, and the mean proportion of *Prevotella* reached the highest 24 h after membrane rupture. Both *Methylobacterium* and *Prevotella* are gram-negative bacilli and have been reported to contribute to some clinical infections^15,16^. Besides, of all the newborn diagnosed of neonatal infection, only one baby’s blood culture was positive of known common pathogen. Whether the elevated WBC or CRP of other newborn were caused by components of the microbiota need further investigation by 16S rRNA genes. Future studies could focus on their association with fetal or neonatal infections.

The LEfSe algorithm showed that *Ruminococcus*, *Paludibaculum*, *Lachnospiraceae*, *and Prevotella* were detected earlier in the fetal membrane or amniotic fluid and then detected in cord blood or maternal peripheral blood, which was consistent with the ascending infection model. Fetal membranes constitute a barrier against bacterial invasion of the amniotic cavity. Following premature rupture of membranes, which was often preceded by cervical dilation, loss of integrity of the membranes directly exposed the amnionic epithelia surface to the endogenous bacterial microbiome of the cervix. Factors including loss of amniotic fluid with its antibacterial properties, increased physical proximity between the cervix and the product of gestation due to loss of amniotic fluid and negative pressure following uterine contractions all contributed to the spread of the microbiome as a “biofilm” along the amnionic epithelia surface^17^. All four bacteria we listed were anaerobes, and their effect on neonates has yet to be explored.

However, it was also found that *Rhodobium, Christensenellaceae, Nonomuraea, Thauera, and Pseudonocardia* were detected in cord blood or maternal blood earlier than in fetal membrane and amniotic fluid, which was different from the ascending pattern of microbiomes. The reason might be that these microorganisms reached the fetus through the hematogenous pathway and reversely seeded into the amniotic fluid from the fetus. There have been an increasing number of reports supporting hematogenous invasion of microbes in intrauterine infection in recent years. Riggs et al. found that the hematogenous spread of *Mycoplasma pulmonis* to the rat fetus can occur without amniotic fluid infection and indicated that the fetus itself can spread the amniotic fluid with microbes^18^. Mendes et al. found that *Streptococcus mutans* could migrate from saliva to maternal peripheral blood and cord blood and that tooth brushing increased *S. mutans* detection in blood samples^19^. However, the mechanism by which oral microbial species move from gravidae to the fetus is not yet fully understood. In addition, Fardini et al. found that after intravenous injection of gingival plaque samples, some species selectively had a higher prevalence in the placenta than in the oral cavity, which indicated that sometimes the translocation was species specific^20^. Among the bacteria discovered in our study, *Pseudonocardia carboxydivorans* was isolated from human cerebrospinal fluid (CSF) from individuals suffering from meningitis, indicating its relationship with infectious diseases, which should be further studied^21^. Ascending infections should be alerted among women with PROM, which is commonly recognized. However, any systemic maternal infection before term could trigger the onset of labor, and rupture of membranes may be a result of infection instead of the causes^3^. Our study demonstrated that the hematogenous pathway of infection should also be considered in PROM patients. However, the specific microorganisms involved and their morbific abundance or duration are poorly understood. Future studies will focus on the associations between these microorganisms and fetal or neonatal diseases. If such a link is revealed, it may allow targeting of neonatal investigation and treatment.

Changes in the microbiomes of maternal, intrauterine, and fetal environments after membrane rupture have rarely been reported in previous studies. Further investigation to identify how each type of bacteria may be clinically relevant with maternal or fetal infection could provide evidence for clinical interventions for PROM patients, including antibiotic use or earlier induction of labor after membrane rupture for term or near term patients. *Methylobacterium, Halomonadaceae* and *Prevotella* could be potential targets for the study of fetal or neonatal infection. In addition, microbiomes may spread in the hematogenous pathway, suggesting the importance of recessive systemic maternal infections in PROM patients.

In our study, 16S rRNA gene amplicon high-throughput sequencing was used to compare the microbiome diversity and signature in maternal, intrauterine, and fetal environments of different durations following membrane rupture, which enabled researchers to further identify potential intrauterine-related bacteria and their possible origins. However, our study has several limitations. The primary limitation was that the blood and intrauterine samples might be more heavily influenced by contaminants produced during the experiment^22,23^. To reduce the impact of this problem, we used the LEfSe algorithm and Kruskal‒Wallis test to minimize its impact. During comparisons, the LEfSe algorithm was set to identify microbes that were significantly enriched in the period than in all other periods of membrane rupture, which may result in the absence of some microorganisms due to the algorithm. Besides, information of the pathological examination of placenta, the number of digital examinations were lost, and amniotic fluid has been sampled only in case of c-section, which may cause bias. What’s more, part of samples was collected from preterm patients, the difference of gestational age between the group of PROM < 6 h and the group of PROM > 48 h could also cause bias. Last one, although the baseline characteristics of women showed that the rate of antibiotic administration was the same in different groups, however, type of antibiotic administered was not totally the same, which could also cause bias. Further large-scale prospective studies were needed to better investigate these problems.

In summary, we found that the diversity of the fetal membrane microorganisms increased significantly after 12 h of membrane rupture, while the diversity of the amniotic fluid microbiome changed after 24 h. Methylobacterium, Halomonadaceae and Prevotella in cord blood changed significantly between the five groups. Source tracking of microorganisms showed that in addition to the common ascending pattern of infection, the hematogenous pathway of intrauterine infection should also be considered in PROM patients.

## Methods

### Study population

This was a secondary analysis of a case–control study to compare the microbiome signature in the maternal, intrauterine, and fetal environment in women who experienced preterm birth and term birth of the Obstetrics and Gynecology Hospital of Fudan University between March 2014 and December 2014. The eligibility criteria were as follows: (1) singleton birth; (2) gestational week of birth ≥ 28 weeks; (3) the successful acquisition of cord blood for test. The exclusion criteria were as follows: (1) women with demonstrated infection or fever before labor; (2) women with a history of antibiotic use one month before PROM; (3) twin pregnancy; (4) elective cesarean section; (5) cases with missing data. All participants received similar obstetrical care.

After consent was obtained, clinical information was extracted directly from the medical records. Maternal clinical metadata included maternal age, times of gestation and parity, gestational weeks at delivery, maternal comorbidities, temperature during labor, the results of blood count assay (including white blood cell [WBC] and neutrophilic granulocyte [N] percent), duration of membrane rupture, the presence of maternal infection during delivery, the presence of meconium stained amniotic, intrapartum antibiotic administration and mode of delivery. The neonatal outcomes we collected included Apgar scores at 1 and 5 min, admission to the neonatal ward, neonatal infection, and perinatal mortality. Neonatal infection was defined as follows: the blood test of newborn showed that WBC ≥ 50 × 10^9^/L and/or CRP (C-reactive protein) ≥ 20 mg/L within 12–24 h after delivery; WBC ≥ 30 × 10^9^/L and/or CRP ≥ 8 mg/L within 24–48 h after delivery; or the blood culture (before the administration of antibiotics) of newborn was positive. Gestational age was confirmed by first trimester sonogram. The duration of membrane rupture was recorded as the time from the beginning of membrane rupture to the delivery of the newborn. Maternal infection was considered when maternal temperature is greater than 38 °C in addition to two other signs (uterine tenderness, maternal or fetal tachycardia and foul/purulent amniotic fluid)^24^. Screening test of Group B streptococcus was carried out for women at 35 gestational weeks and screening tests of mycoplasma, chlamydia, gonococcus and other common bacteria of cervical secretions were carried out for PROM women at the administration of the labor room. Antibiotics such as second-generation cephalosporin or clindamycin (for patients allergic to penicillin) were given at the presence of suspected maternal infection, preterm birth or group B streptococcus positive. Admission to the neonatal ward was at the discretion of experienced neonatologists. According to the duration of membrane rupture, five groups were established (< 6 h, 6 to < 12 h, 12 to < 24 h, 24 to < 48 h and ≥ 48 h).

### Ethics statement

This study received ethical approval from the Obstetrics and Gynecology Hospital of Fudan University (Ethical Review of Obstetrics and Gynecology [2013] No. 26). Informed consent was received from all participants. All methods were performed in accordance with the relevant guidelines and regulations.

### Sample collection

Maternal peripheral blood samples were collected from participants after enrollment in the study. Amniotic fluid (during C-section), placenta, and cord blood samples were collected following delivery. Following delivery, the placentas were placed on a sterile operating platform. A total of three 0.5 × 0.5 cm chorion samples were collected from each placenta. Before collecting the membrane, sterile forceps were used to remove the amnion to decrease potential contaminants. Amniotic fluid was sampled under sterile conditions after direct visualization of the intact amniotic bag through the uterine incision. Umbilical vein cord blood was collected under sterile conditions immediately after delivery and placed into Ethylene diamine tetraacetie acid (EDTA) anticoagulant tubes. All the samples were immediately placed at − 20 °C and then transferred to a − 80 °C freezer within 48 h for storage until DNA extraction.

### DNA extraction, PCR amplification, quantification, and sequencing

DNA was extracted under strict sterile conditions in each sample by the standard protocol from the QIAamp DNA Blood Mini and QIAamp DNA Mini Kits (Qiagen, Hong Kong). All extracted DNA samples were stored at − 20 °C. The V3–V4 region of the 16S rRNA gene was amplified by polymerase chain reaction (PCR) from the microbial genome DNA using appropriate primers (forward primer: 5′-ACTCCTACGGGAGGCAGCAG-3′; reverse primer: 5′-GGACTACHVGGGTWTCTAAT-3′). The PCR products were detected using dual-indexing amplification and then normalized, pooled, and sequenced using an Illumina MiSeq desktop sequencer (250 bp and 300 bp paired-end run)^25^. The PCR conditions were 50 °C for 2 min, 95 °C for 10 min, 95 °C for 15 s, 56 °C for 30 s, and 72 °C for 1 min, repeated for 40 cycles, and then 72 °C for 10 min.

Sequence read processing was conducted using quantitative insights into microbial ecology (QIIME) (version 1.9.0, http://bio.cug.edu.cn/qiime/) and included additional quality trimming, demultiplexing, and taxonomic assignments. Profiling of the microbiota was conducted using phylogenetic investigation of communities by reconstruction of unobserved states (PICRUSt) based on the 13 August 2013 Greengenes database. The output file was further analyzed using the Statistical Analysis of Metagenomic Profiles (STAMP) software package v 2.1.3^26,27^. The raw sequencing paired-end reads were overlapped to form contiguous reads. Trimmomatic and FLASH software were used for quality control and filtering. The sequences were then aggregated to OTUs by Usearch 7.143 (http://qiime.org/) based on 97% pairwise identity using QIIME’s open reference OTU picking strategy. Taxonomic classification of the representative sequence for fungal OTUs was conducted using the Ribosomal Database Project classifier (Release 11.1 http://rdp.cme.msu.edu/) against the fungal internal transcribed spacer (ITS) database in Unite (Release 5.0 http://unite.ut.ee/index.php). Chimeric OTUs were detected using UCHIME (version 4.2.40, http://drive5.com/usearch/manual/uchimealgo.html) and removed from the OUT Table^28^.

### Statistical analysis

Categorical data were compared using the χ^2^ test. Continuous data were compared using Student’s *t* test. Alpha diversity was analyzed by the methods of Sobs indices^29^. Wilcoxon’s test was used to detect significant features among the groups. Beta diversity was calculated using analysis of similarity in Bray‒Curtis distance. The Kruskal‒Wallis test and the LEfSe algorithm were used to detect significant features that differentiated the groups. The threshold of the LEfSe score for discriminative features was 2.0. *P* < 0.05 was considered statistically significant.

## Data Availability

The data analyzed during this study are available in the NCBI SRA repository under BioProject accession number PRJNA977102 [https://www.ncbi.nlm.nih.gov/bioproject/PRJNA977102].

## Abbreviations

PROM: Premature rupture of membranes
pPROM: Preterm premature rupture of membranes
LEfSe: Linear discriminant analysis effect size
OUT: Operational taxonomic unit
CSF: Cerebrospinal fluid
WBC: White blood cell
N: Granulocyte
CRP: C-reactive protein
EDTA: Ethylene diamine tetraacetie acid
PCR: Polymerase chain reaction
QIIME: Quantitative insights into microbial ecology
PICRUSt: Phylogenetic investigation of communities by reconstruction of unobserved states
ITS: Internal transcribed spacer
